# Mapping deep peat carbon stock from a LiDAR based DTM and field measurements, with application to eastern Sumatra

**DOI:** 10.1186/s13021-020-00139-2

**Published:** 2020-03-23

**Authors:** Ronald Vernimmen, Aljosja Hooijer, Rizka Akmalia, Natan Fitranatanegara, Dedi Mulyadi, Angga Yuherdha, Heri Andreas, Susan Page

**Affiliations:** 1 Inland Water Systems Unit, Deltares, P.O. Box 177, 2600 MH Delft, The Netherlands; 2 Present Address: Data for Sustainability, 4571 AK Axel, The Netherlands; 3 PT Alas Rawa Khatulistiwa, Jakarta, Indonesia; 4 Geodesy Research Group, Institute of Technology Bandung (ITB), Jl. Ganesha 10, Bandung, Indonesia; 5 Centre for Landscape and Climate Research, School of Geography, Geology and the Environment, University of Leicester, Leicester, LE1 7RH UK

**Keywords:** Below-ground carbon stock, Peat, Peat thickness, Lowland, Sumatra, LiDAR, ICESat-2 DTM

## Abstract

**Background:**

Reduction of carbon emissions from peatlands is recognized as an important factor in global climate change mitigation. Within the SE Asia region, areas of deeper peat present the greatest carbon stocks, and therefore the greatest potential for future carbon emissions from degradation and fire. They also support most of the remaining lowland swamp forest and its associated biodiversity. Accurate maps of deep peat are central to providing correct estimates of peat carbon stocks and to facilitating appropriate management interventions. We present a rapid and cost-effective approach to peat thickness mapping in raised peat bogs that applies a model of peat bottom elevation based on field measurements subtracted from a surface elevation model created from airborne LiDAR data.

**Results:**

In two raised peat bog test areas in Indonesia, we find that field peat thickness measurements correlate well with surface elevation derived from airborne LiDAR based DTMs (R^2^ 0.83–0.88), confirming that the peat bottom is often relatively flat. On this basis, we created a map of extent and depth of deep peat (> 3 m) from a new DTM that covers two-thirds of Sumatran peatlands, applying a flat peat bottom of 0.61 m +MSL determined from the average of 2446 field measurements. A deep peat area coverage of 2.6 Mha or 60.1% of the total peat area in eastern Sumatra is mapped, suggesting that deep peat in this region is more common than shallow peat and its extent was underestimated in earlier maps. The associated deep peat carbon stock range is 9.0–11.5 Pg C in eastern Sumatra alone.

**Conclusion:**

We discuss how the deep peat map may be used to identify priority areas for peat and forest conservation and thereby help prevent major potential future carbon emissions and support the safeguarding of the remaining forest and biodiversity. We propose rapid application of this method to other coastal raised bog peatland areas in SE Asia in support of improved peatland zoning and management. We demonstrate that the upcoming global ICESat-2 and GEDI satellite LiDAR coverage will likely result in a global DTM that, within a few years, will be sufficiently accurate for this application.

## Background

Coastal peatlands cover some 10% of the land area in much of SE Asia and are subject to a range of environmental issues following deforestation and drainage in recent decades, including peat oxidation and fires that result in haze and major carbon emissions [1–5], biodiversity loss [6–9], and increased flooding risk following land subsidence [10–12]. Reducing carbon emissions from peatlands in this region has been recognized as a key factor in reducing global peatland emissions, aiming to mitigate climate change [1, 13, 14].

Peat consists of partially decomposed plant material, which will accumulate when net primary production exceeds the rate of decomposition. In temperate and boreal climates where most peat occurs [15], decomposition is inhibited by anaerobic conditions [16] and cold temperatures [17]. In the tropics decomposition is inhibited by anaerobic conditions [18] and more recalcitrant organic matter [13, 19, 20]. While large areas of tropical peat are also found in South America and Central Africa [2, 21–23], SE Asia hosts an estimated 38% of both tropical peat area and volume [24]. Indonesia is the country with the most tropical peat with estimates of below-ground carbon stock ranging between 28.1 and 57.4 Pg C [2, 25, 26].

Since peat is mostly water (80–> 90%) and organic matter (of which some 51–56% is carbon; [2, 27, 28]), it is not a stable material like most other soils that have a high mineral content. When dried, peat shrinks, oxidizes and can easily burn. Shrinkage and oxidation as well as peat combustion result in land surface subsidence [29–36] that often results in flooding (e.g. [37–40]). In much of the world, remaining intact peatlands have therefore been protected, and drained peatlands are being carefully managed and, in some cases, restored, to mitigate these impacts (e.g. [41]).

In Indonesia, peatlands in their natural state are covered by tropical rainforest with very high biodiversity [42, 43]. Limited access and poor conditions for agriculture and road construction have long kept these areas undisturbed by human activities. Peat of depths over 2 m was considered unsuitable for development until the 1990s [44–46] and was protected by Indonesian law. However, since then, extensive areas of peatland, including those with peat exceeding a thickness of 3 m, have been deforested and drained at a rapid rate, with only 29.1% of peatland in Sumatra and Kalimantan remaining forested by early 2015 and only 6.5% classed as undisturbed primary forest [47]. A further major reduction in peatland forest cover occurred during late 2015 when an estimated 0.7 to 1.2 Mha of peatland in Sumatra and Kalimantan were burnt [48, 49].

Following the devastating fires of recent years, especially 2014 [4] and 2015 [5], the Indonesian Government and some plantation companies have made efforts to limit the deforestation and drainage of peatland, and to raise water levels in some peatlands that burnt in 2015 [50]. This may provide an opportunity to prevent further peat loss and conserve the limited areas of remaining peat swamp forest. However, this will require conservation zoning based on reliable peat thickness maps. Existing peat thickness maps for Indonesia are inaccurate [26, 51] and in some areas contested. The Indonesian Peat Prize process was started to improve maps [52] but creating such a map to a level of accuracy that suits all purposes will likely take some years.

For land use planning at the landscape scale, accuracy requirements in peat thickness mapping are lower than for detailed management plans at the plot scale. For identification of peatland areas in Indonesia most urgently requiring conservation (where forest is still present) or restoration (where peatland is already cleared of forest and drained), it may be sufficient to prioritize locations where the peat is over 3 m in thickness and presents the highest carbon stock per unit area. Furthermore, several Indonesian regulatory and policy measures at the national, sectoral and local levels require deep peat (> 3 m) to be protected and conserved. For an overview of these regulations see [53]. It is also the deeper peat areas that support any remaining peat swamp forest; hence in these situations, both high carbon stock and high biodiversity can be prioritized. This is not to say that shallower peats (< 3 m) do not play a role in carbon storage and release, but in Indonesia most shallow peats have been subject to a more intense degree of land use pressure and most are under some form of agriculture. These highly modified and degraded peats would require a much greater range of interventions (ecological and socio-economic) to bring about successful restoration outcomes.

In this paper, we present a model of peat cover and thickness for the deep peat (> 3 m) areas of the eastern Sumatra lowlands. The four objectives of the study are (i) to present a method to model extent and thickness of deep peat (> 3 m) for the study region using a LiDAR-based DTM (ii) to validate the accuracy of the model using field measurements; (iii) to use the map of deep peat to calculate peat carbon stocks and to highlight priority locations for peatland and forest protection; and (iv) to investigate whether such assessment is possible with the satellite LiDAR data that is now becoming available globally.

## Methods

### Study area

The study focuses on the eastern Sumatra lowland that has the largest mapped concentration of peatland in Indonesia [54, 55], at 5.7 Mha in the Provinces of Riau, Jambi and South Sumatra alone [56]. Peatlands in this area have been subject to especially high rates of deforestation and plantation development since 2000 [57, 58], but still host some large protected peat swamp forest areas, such as the Giam Siak Kecil—Bukit Batu Biosphere Reserve, Kerumutan Wildlife Reserve and Berbak and Sembilang National Parks [59]. While Sembilang National Park is mostly dominated by mangrove forests without large peat domes, it does contain smaller domes and borders Berbak National Park, both host peat swamp forest [60]. Indonesian national and local governments and several major plantation companies that are active in this region have developed peat swamp forest conservation programs [61–68] that will benefit from reliable maps of peat and forest conditions. We also included a small area at 10–40 km from the coast in West Kalimantan, Kubu Raya, in this study, to demonstrate the broader validity of the methods beyond Sumatra.

### DTMs from airborne LiDAR data

We applied a DTM created from airborne LiDAR data for eastern Sumatra [69] and for the Kubu Raya validation area in West Kalimantan [70], with an estimated overall vertical accuracy between 0.25 and 0.61 m [69].

### Peat thickness field measurements collected in validation areas

**Fig. 1:**
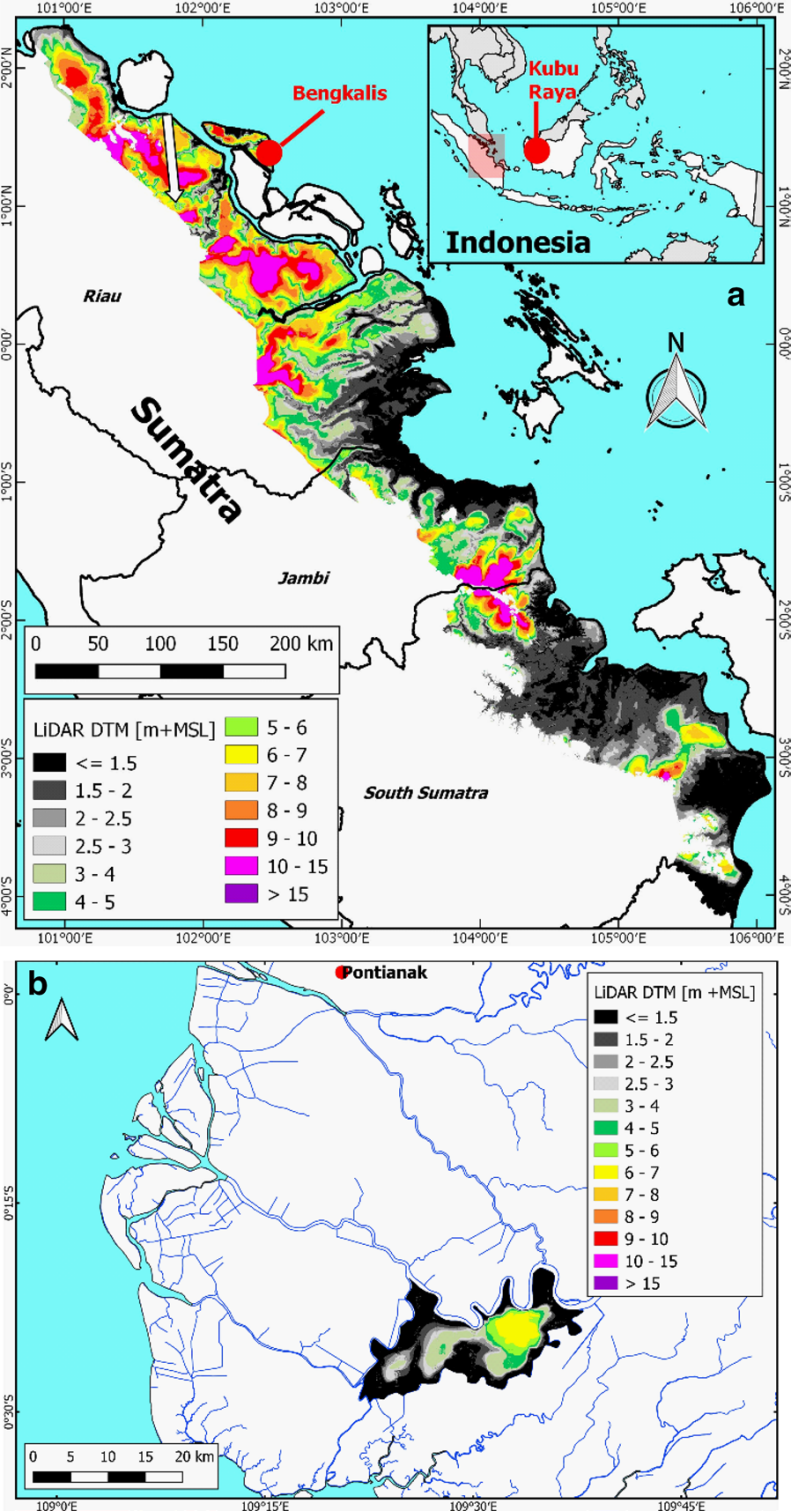
**a** Eastern Sumatra lowland DTM area [69] where peat was mapped in this study. Indicated with red dots the areas where the mapping methodology was validated. The ICESat-2 profile location over Giam Siak Kecil—Bukit Batu Biosphere Reserve shown in Fig. 12 is shown with the white arrow. **b** The LiDAR DTM for the Kubu Raya study area

Field surveys conducted in 2017 in the Bengkalis (54,133 ha) and Kubu Raya (23,681 ha) method validation areas applied a high accuracy survey protocol [70, 71]. Peat thickness, i.e. an organic top soil horizon of over 0.5 m thickness, was measured (Fig. 1) by surveyor teams using Edelman type augers, along straight line transects perpendicular to rivers, streams and sometimes canals and going up the peat dome slopes. Measurements were replicated at least twice at 5 m distance, until the difference between peat thickness values was less than 1 m, with the interface between the peat and underlying mineral soil being photographed for later verification. The average value derived from approved measurements was used in further analyses.

### Measuring peat bottom elevation relative to mean sea level

The peat thickness measured in the field was subtracted from the DTM surface elevation to obtain the peat bottom level, i.e. the interface between the peat and the underlying mineral sediment, at the measurement location, for all peat thickness measurements considered in this study.

### Creating peat thickness models

**Fig. 2:**
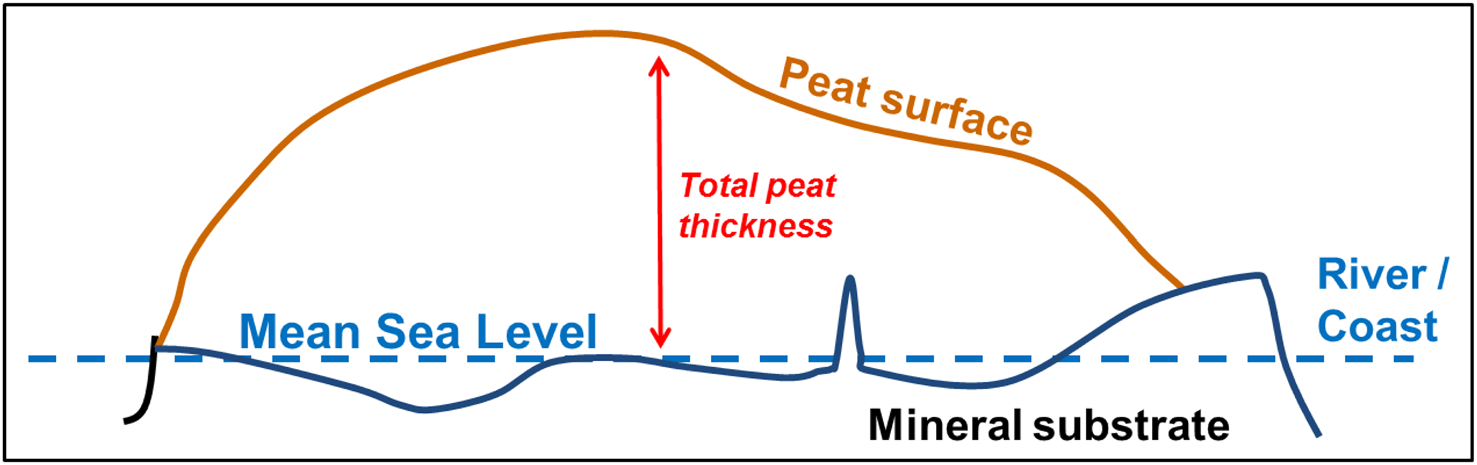
Illustration of how peat thickness is determined as the difference between the peat surface and depth of the peat bottom (interface with the mineral substrate)

Peat thickness models were created using airborne LiDAR based peat surface DTMs and average peat bottom levels derived from peat thickness field measurements. Peat thickness was determined as the difference between peat surface and peat bottom, as illustrated in Fig. 2.

References [70, 71] demonstrated the application of three different peat mapping methods to two validation areas, Bengkalis in Riau (Sumatra) and Kubu Raya in West Kalimantan. The first method involved the creation of a peat bottom model by interpolation of a peat bottom surface model between measurement points and contours that were manually drawn and subtracting this model from the DTM. The second method assumed a flat horizontal peat bottom surface determined from the average of available data, while the third method applied a regression equation between DTM and available peat thickness data. Considering that the three resulting peat thickness models were nearly identical [70, 71], the simplest method, assuming a flat horizontal peat bottom surface as determined from the average of available data, was applied in this study. The peat bottom level was subtracted from the peat surface elevation map to generate a peat thickness map.

### Mapping peat extent in validation areas

**Fig. 3:**
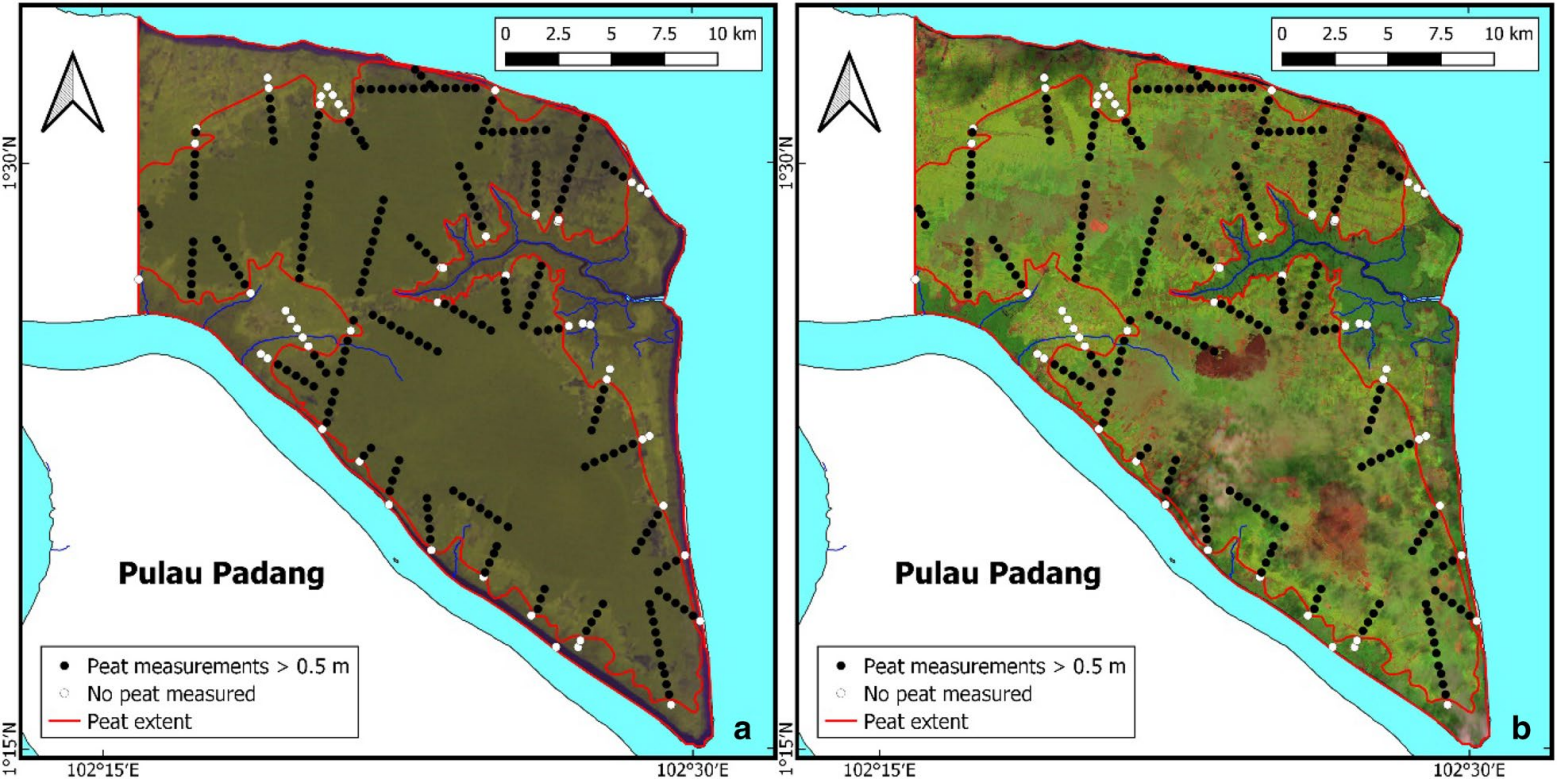
Peat extent boundary (red line) in the Bengkalis study area that was delineated based on visual interpretation of (**a**) background RGB composite (spectral bands 6-7-5) Landsat-1 image of 5 October 1972 and peat thickness measurements (black and white dots). For comparison, the peat extent boundary is also shown on (**b**) Sentinel-2 background RGB image of 4 August 2016 (spectral bands 11-8-5). Note that nearly all peat was still forested in 1972

Peat extent in the Bengkalis peat mapping method validation area was identified through visual interpretation of a composite Landsat-1 image of 5 October 1972 (i.e. when the area was still largely forested and peat extent could be discerned from vegetation patterns) [71] (Fig. 3). But whereas historical remote sensing imagery is often useful for visual interpretation of the peat extent from vegetation patterns, we found this not to be the case for the Kubu Raya area. Instead, we mapped the likely peat extent by applying the 2 m +MSL contour line determined from the airborne LiDAR based DTM [70]. In both areas the resulting peat extent was then further refined based on field measurements of peat presence and absence.

### Validating modelled peat thickness

For the Bengkalis and Kubu Raya validation areas, peat thickness maps were created by subtracting a uniform peat bottom level from the DTM at 100 m spatial resolution. The uniform peat bottom was calculated as the average of peat bottom values determined from the DTM at 100 m spatial resolution and individual peat thickness measurements, for both areas separately. Peat thickness field measurements were then compared with modelled peat thickness at the same locations to determine the accuracy of the resulting peat thickness models. Average peat thickness for both models was calculated as the arithmetic mean of the modelled peat thickness for each of the individual 100 m grid cells.

### Estimating peat bottom elevation for eastern Sumatra

From a larger database of existing field measurements [51], adding unpublished data from recent surveys, a set of 2446 measurements was extracted for peat > 3 m thickness, located within the maximum extent of deep peat as estimated in this study, and the arithmetic mean was calculated. 2198 (89.9%) of the measurements were collected after 2010, and of the 2446 measurements, Riau had the most measurements 1587 (64.9%), followed by South Sumatra and Jambi, with 487 (19.9%) and 372 (15.2%) measurements, respectively. The selected data were collected by 45 different sources between 2000 and 2017. It was found that in areas where multiple datasets were available for comparison, the data often showed considerable differences, especially for older data that were collected before GPS provided accurate locations. This illustrates the difficulty in conducting robust peat thickness surveys, at the large scale and under often difficult field conditions by sometimes untrained teams with often limited field supervision by experts, applying different protocols, and using different auger types [51]. Taken as a whole however, and excluding data collected before 2000, the compilation dataset does provide sufficient basis for estimating average peat bottom elevations over large areas.

### Creating a map of deep peat for eastern Sumatra

Deep coastal peat (peat thickness > 3 m) was mapped in the eastern Sumatra lowland within the available DTM extent (Fig. 1) and within the latest official peat extent as published by the Indonesian Ministry of Agriculture [56], excluding some smaller peat areas in river valleys surrounded by mineral uplands further inland where the peat bottom is expected to be no longer flat but sloping. Peat thickness was mapped by subtracting average peat bottom values determined from 2446 peat thickness measurements > 3 m from the DTM. Because this method was validated mostly with measurements of deep peat in Bengkalis and Kubu Raya, and is likely to be less reliable for areas of shallow peat, only peat thickness over 3 m is shown in the resulting peat maps and in carbon stock calculations presented here.

### Calculating deep peat carbon stocks

The amount of carbon stored in a unit volume of peat was estimated by multiplying the bulk density (BD) by the carbon concentration (CC) of dry peat. Since BD and CC are known to spatially vary we have applied peat carbon density values ranging from 0.0545 to 0.0698 g cm^−3^, as derived for different Indonesian provinces from literature by [26], to determine a lower and upper limit of carbon stocks.

## Results

### Peat thickness models for validation areas

**Fig. 4:**
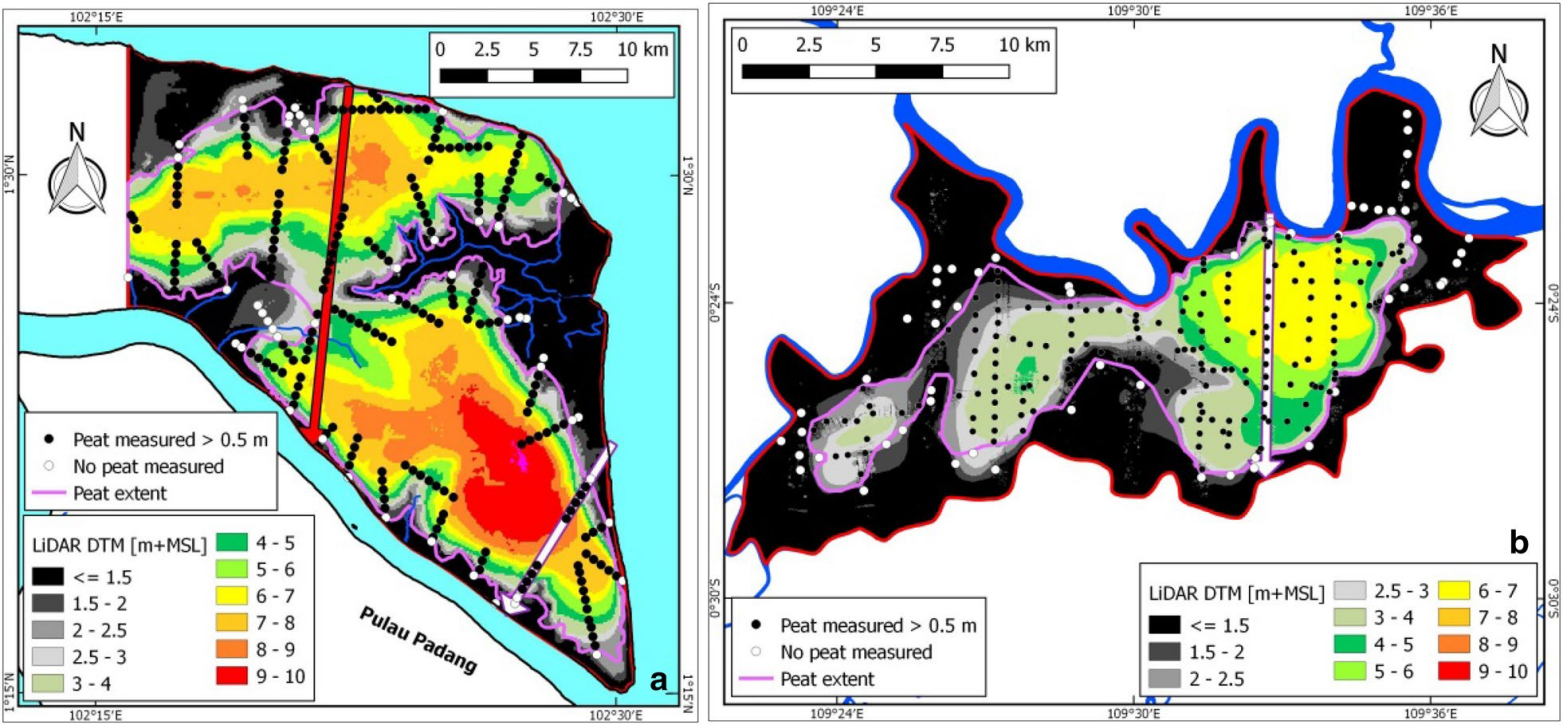
Peat thickness measurement locations in Bengkalis (**a**) and Kubu Raya (**b**) study areas (red lines). In the background airborne LiDAR derived DTMs (Bengkalis: [71]; Kubu Raya: [70]). Locations of cross sections shown in Fig. 6 are indicated with white arrows. The ICESat-2 profile location over Bengkalis shown in Fig. 12 is shown with the red arrow

In total, a dataset of 508 field measurements (excluding replicates) was applied in peat mapping for the Bengkalis and Kubu Raya areas (Fig. 4). Of these, 399 were found to have peat (> 0.5 m), and 53.1% (n = 270) had a peat thickness over 3 m. Average peat thickness was 4.79 m (± 2.17 m st. dev.) and 3.45 m (± 1.60 m st. dev.), respectively (Table 1).

**Table 1:** Peat thickness (PT) measurement statistics for the Bengkalis and Kubu Raya study areas

Statistic	Bengkalis	Kubu Raya
Number of PT measurements > 0.5 m	219	180
Number of PT measurements > 3 m	170	100
Average PT (m)	4.79	3.45
Standard deviation PT (m)	2.17	1.60
Average PB (m +MSL)	0.41	0.30
Standard deviation PB (m)	0.90	0.55
Number of PT measurements > 0.5 m with PB below 0 m +MSL (%)	81 (37.0%)	52 (28.9%)
Number of PT measurements > 0.5 m with PB below 1.5 m +MSL (%)	198 (90.4%)	177 (98.3%)
Number of PT measurements > 0.5 m with PB below 2 m _MSL (%)	216 (98.6%)	179 (99.4%)
Number of PT measurements > 0.5 m within 0.5 m of average PB (%)	72 (32.9%)	109 (60.6%)
Number of PT measurements > 0.5 m within 1 m of average PB (%)	160 (73.1%)	168 (93.3%)
Number of PT measurements > 3 m within 1 m of average PB (%)	128 (75.3%)	92 (92.0%)

Each peat thickness value presents the average of 2 or more replicate measurements

*PB* peat bottom

**Fig. 5:**
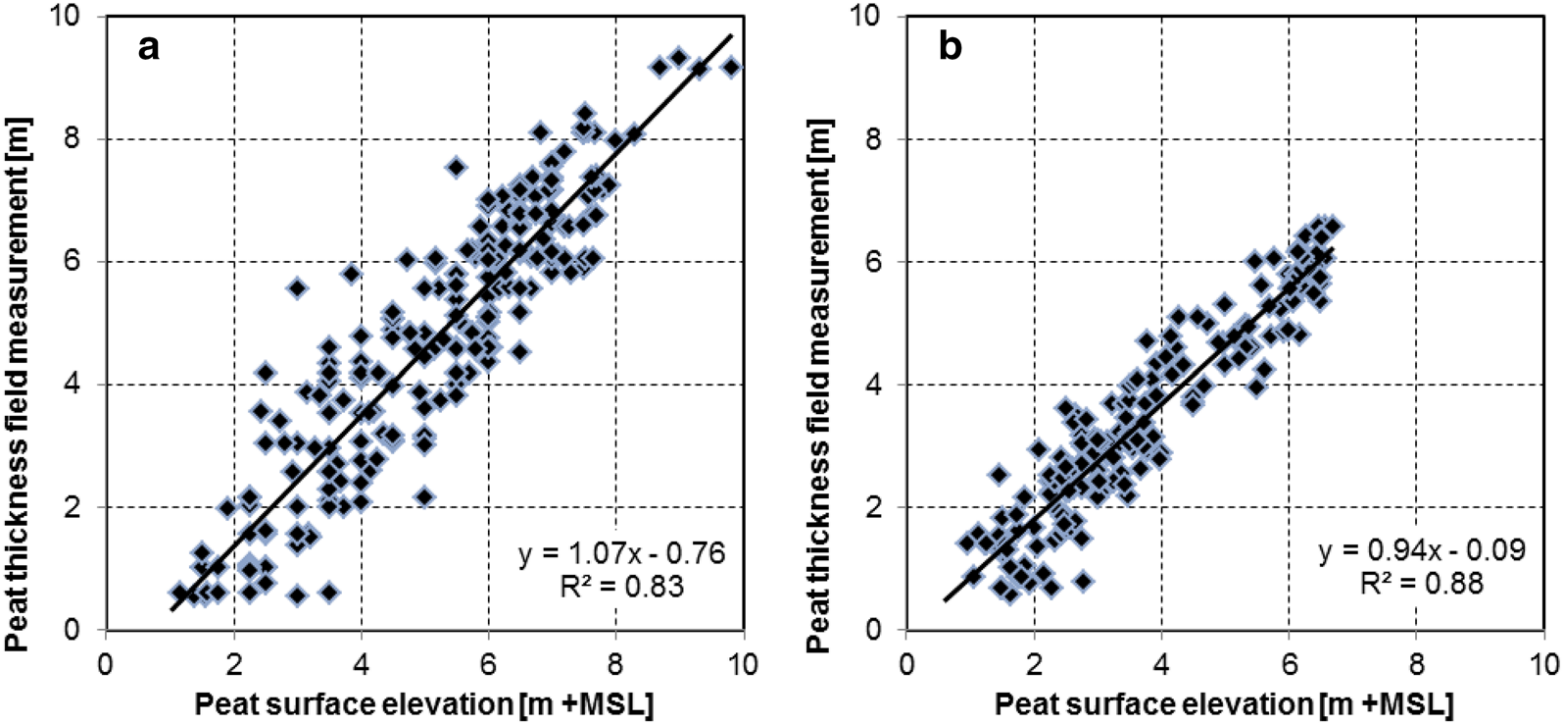
Peat thickness measurements plotted against elevation as determined from the airborne LiDAR DTM for the **a** Bengkalis (n = 219) and **b** Kubu Raya (n = 180) study areas

High R^2^ values of 0.83 and 0.88 between surface elevation and peat thickness were found for the Bengkalis and Kubu Raya study areas, respectively, with regression relations approaching unity (1:1) in both cases (Fig. 5). This confirms that the peat bottom is relatively flat and close to sea level, and that a DTM may be used for peat thickness mapping. In these areas the average position of the peat bottom was found to be 0.41 and 0.30 m +MSL and the peat bottom was below 2 m +MSL for almost all measurements (98.6 and 99.4% of measurements respectively) (Table 1).

**Fig. 6:**
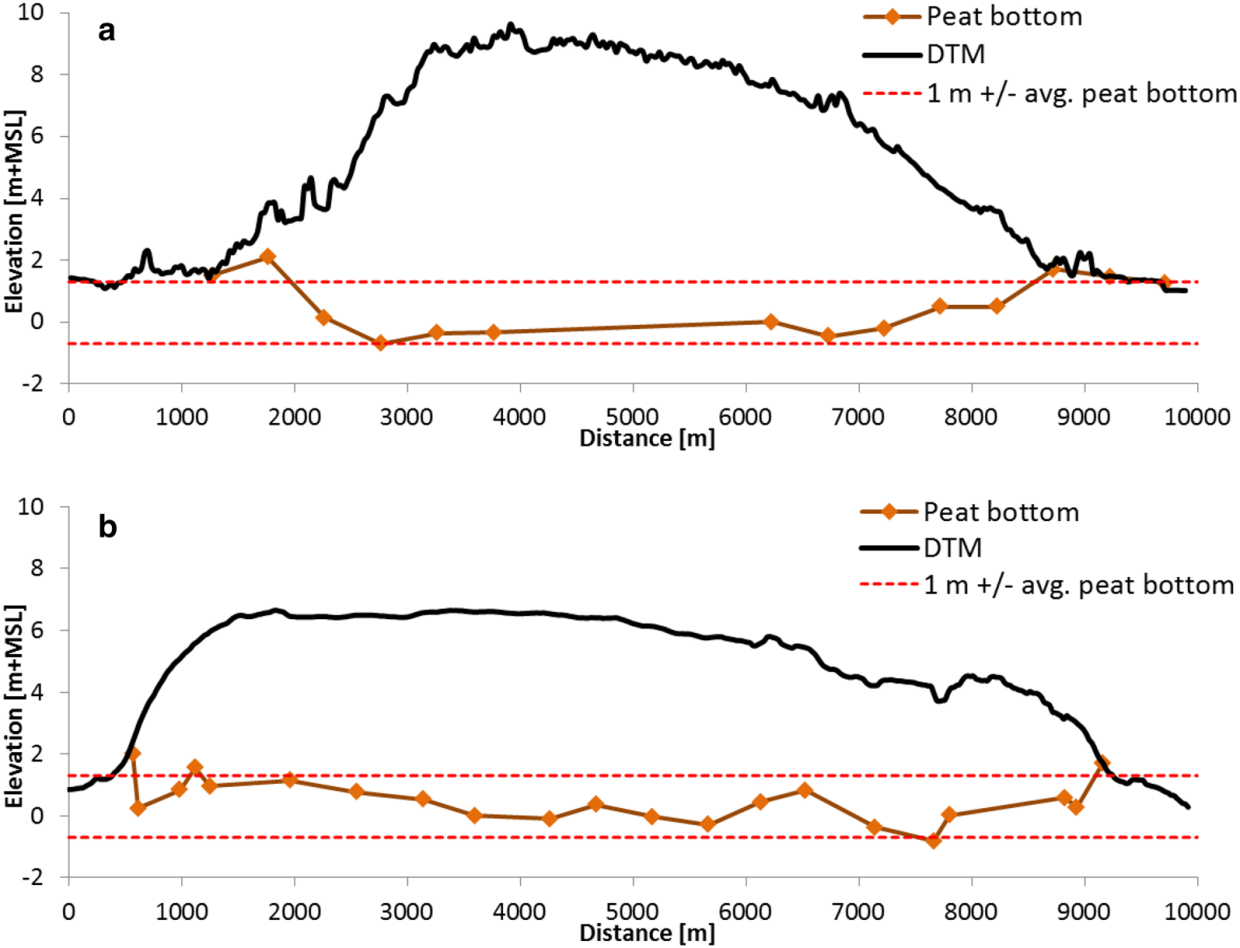
Cross sections over the **a** Bengkalis and **b** Kubu Raya peat domes, showing LiDAR derived surface elevation (DTM) and the peat bottom as derived from field measurements. Locations of cross sections are shown in Fig. 4

When assuming a flat peat bottom throughout the two study areas, 82.2% (n = 328) of the actual individual field measurements of peat bottom elevation were within 1 m above or below the average peat bottom of the two respective study areas and within 81.5% (n = 220) if only peat over 3 m thickness is considered (Table 1; Fig. 6). The overall RMSE is 0.89 m and 0.68 m, for Bengkalis and Kubu Raya, respectively, and 0.87 m and 0.54 m if only peat over 3 m thickness is considered.

**Fig. 7:**
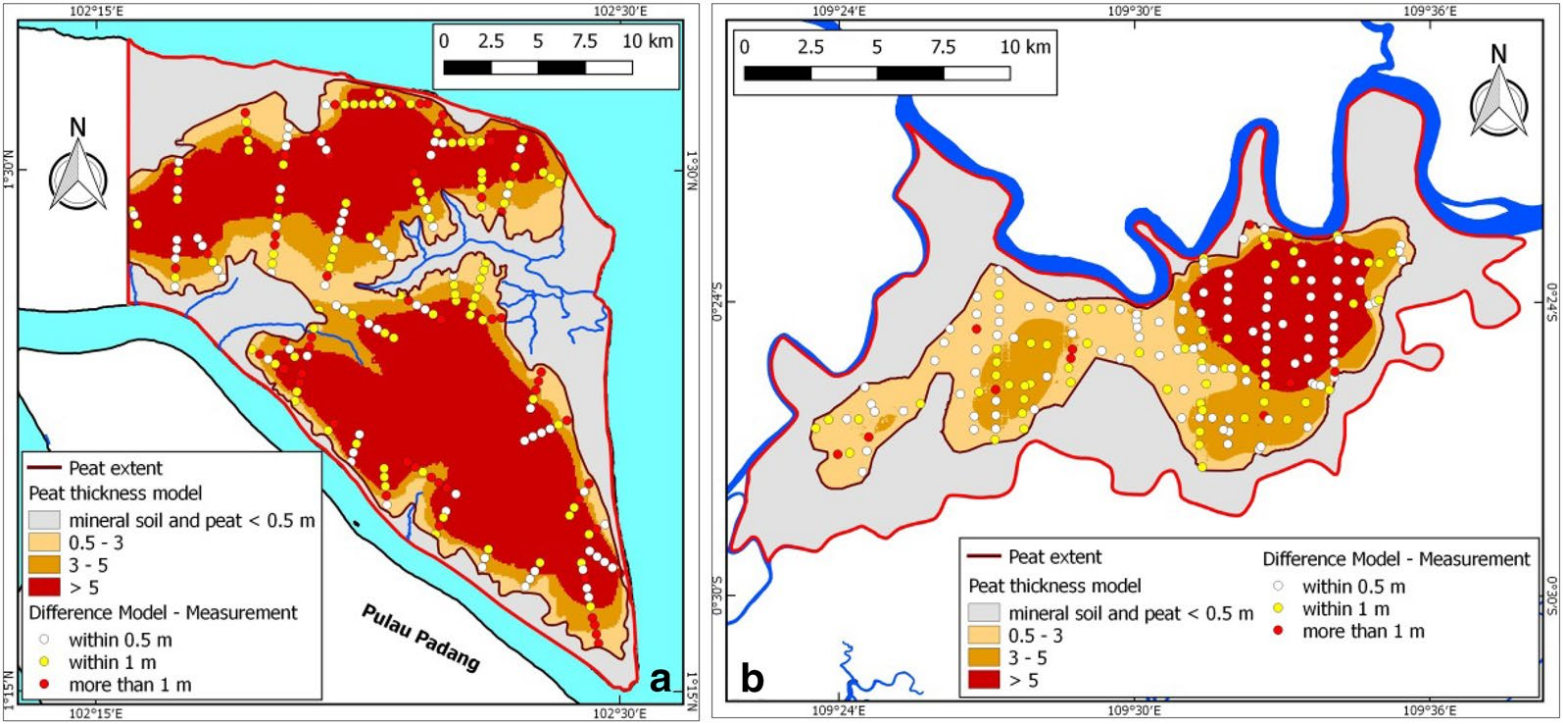
Peat thickness models for **a** Bengkalis and **b** Kubu Raya as derived from airborne LiDAR based DTM and applying a constant peat bottom elevation of 0.41 and 0.30 m +MSL, respectively (Table 1). Peat thickness difference as calculated from the measurements and the peat thickness model is also shown

The resulting peat thickness models for the Bengkalis and Kubu Raya study areas, derived by applying average peat bottom values of 0.41 and 0.30 m +MSL, respectively, are shown in Fig. 7. Of the total peat area (39,074 ha or 72.2% and 10,763 ha or 45.5% of the Bengkalis and Kubu Raya study areas, respectively), 80.5% and 53.6% has peat over 3 m in the Bengkalis and Kubu Raya study areas, respectively, and an overall average peat thickness of 5.39 m and 3.52 m for all peat or 6.26 m and 4.76 m for the peat area over 3 m. Total peat volume was calculated to be 2.1 km^3^ and 0.38 km^3^ for Bengkalis and Kubu Raya, respectively, and 2.0 km^3^ and 0.27 km^3^ for peat over 3 m.

### Measured peat thickness and peat bottom level for eastern Sumatra

**Fig. 8:**
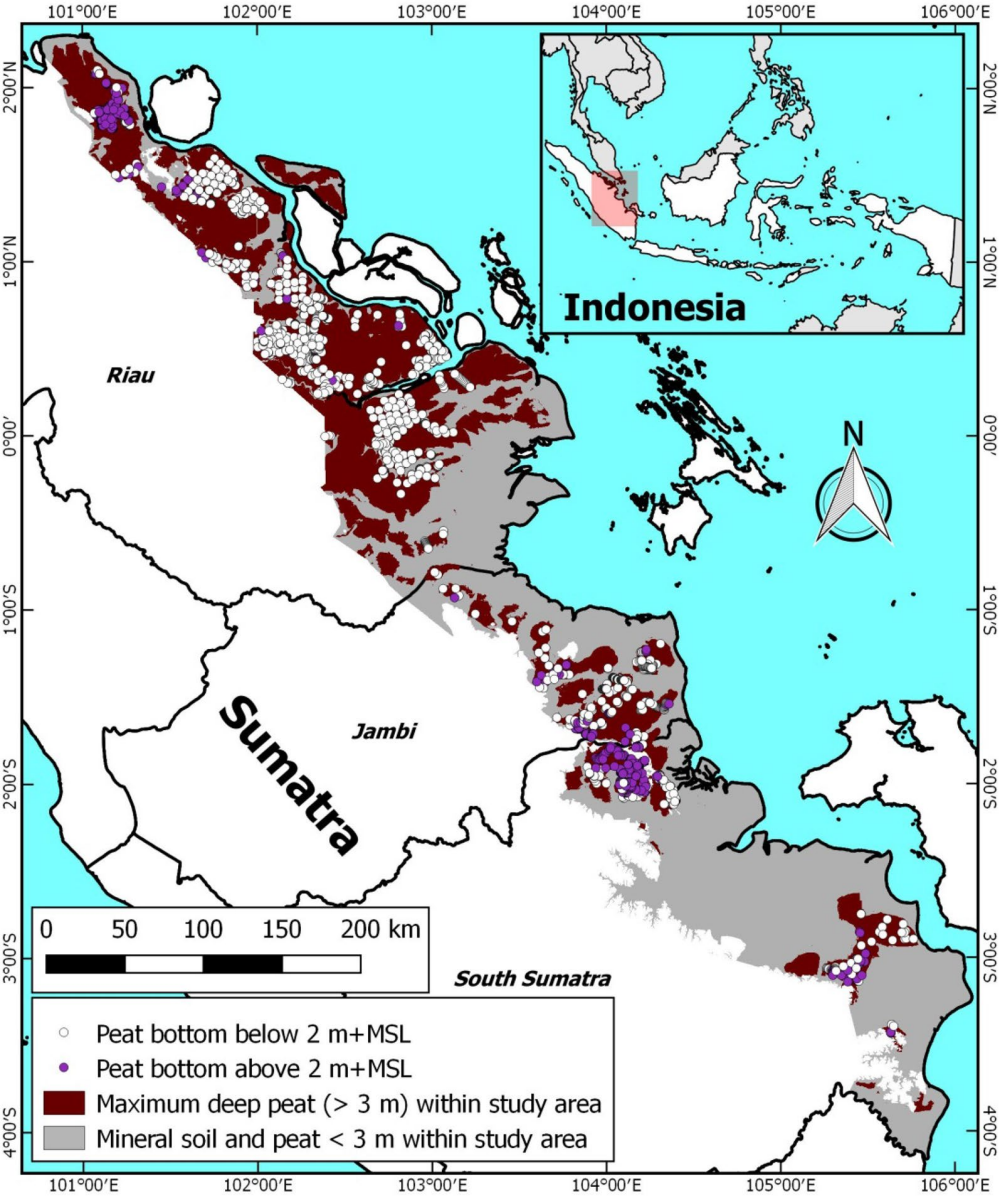
Maximum deep peat areas in eastern Sumatra, mapped by subtracting a flat peat bottom of 0 m +MSL from the airborne LiDAR based DTM for eastern Sumatra (Fig. 1). Location of peat bottom measurement locations are shown with the white (peat bottom below 2 m +MSL) and purple (peat bottom above 2 m +MSL) dots. The DTM extent applied in the analysis is indicated by coloured (brown and grey) areas

The compilation dataset of 2446 existing deep peat thickness measurements in eastern Sumatra yields an average peat thickness of 6.78 m (± 2.40 m st. dev.) and an average peat bottom of 0.61 m (± 2.36 m st. dev.) +MSL, with 40.6% and 74.9% of measurements being below 0 and 2 m +MSL, respectively (Table 2; Fig. 8).

**Table 2:** Summary statistics of peat thickness (PT) measurements over 3 m in eastern Sumatra lowland

Province	No. of measurements	Avg. PT (m)	St. dev. PT (m)	Avg. PB (m +MSL)	St.dev. PB (m)	% of measurements with PB below		
						0 m +MSL	1.5 m +MSL	2 m +MSL
Riau	1587	7.10	2.39	0.02	1.91	49.2	81.4	86.7
Jambi	372	7.12	2.71	0.72	2.99	40.3	59.9	67.5
South Sumatra	487	5.47	1.60	2.47	2.18	12.9	30.8	41.9
Total	2446	6.78	2.40	0.61	2.36	40.6	68.1	74.9

*PB* peat bottom

This confirms earlier reports of the peat bottom in tropical coastal peatlands usually being at or close to sea level [72, 73] further supported by evidence shown in cross sections of peat domes in Sarawak [74–82], Riau [29] and Jambi [76, 83]. This is explained by coastal peat development in eastern Sumatra starting from river floodplains and mangroves some 6000 years ago [54, 79, 84] on a nearly flat coastal plain [84, 85].

While there are regional differences in peat bottom (Table 2), we conclude that applying a flat peat bottom at the average value of 0.61 m +MSL yields the best approximation of likely peat thickness that is possible with the data available. Applying a peat bottom at 2 or 0 m +MSL yields an estimate of minimum and maximum peat thickness, respectively.

### Deep peat map for eastern Sumatra

**Fig. 9:**
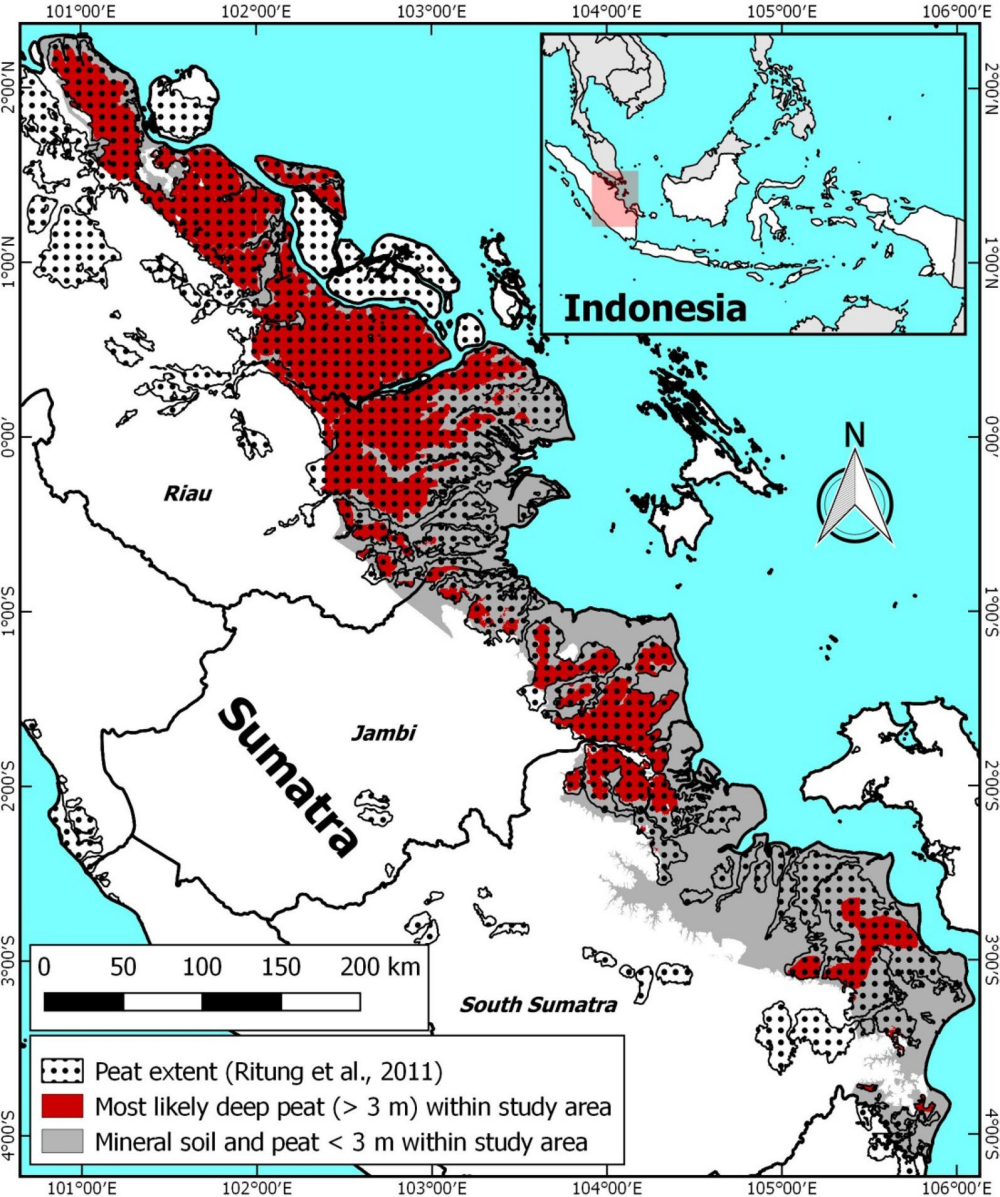
Most likely deep peat areas in eastern Sumatra (Table 1), mapped by subtracting a flat peat bottom of 0.61 m +MSL from the airborne LiDAR based DTM for eastern Sumatra (Fig. 1). Probable peat extent according to [56] is shown as black dots. The DTM extent applied in the analysis is indicated by coloured (brown and grey) areas

The most likely deep peat extent for eastern Sumatra is presented in Fig. 9. The total modelled deep peat area in the eastern Sumatra lowlands ranges between 2.0 and 2.9 Mha, for the applied flat peat bottom range of 0 to 2 m +MSL, respectively. This equals 45.8% to 67.4% of the total probable peat extent (according to [56]) of 4.3 Mha that falls within the DTM study area. Applying the different regional most likely peat bottom values of 0.02, 0.72 and 2.47 m +MSL, for Riau, Jambi and South Sumatra, respectively, yields a deep peat extent of 2.6 Mha (Table 3). This deep peat extent is similar as when applying a most likely peat bottom value of 0.61 m +MSL, which is the average of all measurements in Table 2. The 2.6 Mha mapped most likely deep extent is 60.1% of the area mapped by [56], suggesting that deep peat is more common in this region than shallow peat, and far more extensive than the 33.0% reported by [56] for this same area. Recognizing that previously mapped peat extent is likely to be underestimated by some 10% [25, 51] we conservatively conclude that the area of deep peat in eastern Sumatra is roughly equal or somewhat larger than the area of peat of less than 3 m depth.

**Table 3:** Areas of modelled deep peat, average peat thickness (PT) and below-ground carbon stock in deep peat areas in eastern Sumatra lowland and breakdown per Province, assuming different peat bottom positions

Assumed peat bottom (m +MSL)	Deep peat area (Mha)	Deep peat area (%)	Avg. PT (m)	Below-ground deep peat carbon stock (Pg C)	Below-ground deep peat carbon stock (%)	Deep peat forest cover (Mha)	Deep peat forest cover (%)
	Eastern Sumatra coastal lowland						
0 ‘maximum peat extent’	2.9 (2.6–3.1)	67.4 (60.2–73.9)	6.7 (6.5–6.9)	10.4–13.3 (9.0–15.2)	100.0	1.3 (1.2–1.3)	45.1 (42.4–47.9)
0.61 ‘most likely peat extent’	2.6 (2.0–3.1)	60.1 (47.1–73.9)	6.5 (6.0–6.9)	9.0–11.5 (6.6–15.2)	100.0	1.2 (1.1–1.3)	47.9 (42.4–52.8)
1 ‘extent of peat below high tide limit’	2.4 (2.1–2.7)	53.7 (46.8–60.0)	6.3 (6.1–6.5)	8.2–10.5 (7.0–12.2)	100.0	1.2 (1.1–1.3)	49.3 (46.9–52.0)
1.5 ‘extent of peat above flood limit’	2.2 (1.9–2.4)	51.0 (44.2–57.3)	6.1 (5.9–6.3)	7.2–9.3 (6.1–10.8)	100.0	1.1 (1.0–1.2)	51.3 (48.9–53.8)
2 ‘minimum peat extent’	2.0 (1.7–2.2)	45.9 (40.0–51.8)	5.9 (5.7–6.2)	6.3–8.1 (5.3–15.7)	100.0	1.0 (0.94–1.1)	53.2 (51.0–55.4)
Area weighted (‘most likely peat extent’ per Province)	2.6 (2.1–3.2)	61.4 (48.6–74.6)	6.6 (6.2–7.1)	9.5–12.1 (7.0–15.7)	100.0	1.2 (1.1–1.3)	47.9 (42.5–52.9)
	Riau coastal lowland						
0 ‘maximum peat extent’	2.1 (1.9–2.3)	78.7 (70.8–84.9)	6.8 (6.6–7.1)	7.8–10.0 (6.8–11.3)	75.1 (74.4–75.7)	1.0 (0.95–1.0)	47.5 (45.1–50.1)
0.02 ‘most likely peat extent’	2.1 (1.7–2.5)	77.4 (63.5–91.7)	6.8 (6.3–7.4)	7.7–9.9 (5.9–12.7)	75.1 (72.9–76.3)	1.0 (0.89–1.0)	48.0 (42.6–52.3)
1 ‘extent of peat below high tide limit’	1.8 (1.6–2.0)	66.9 (59.3–74.0)	6.4 (6.2–6.6)	6.2–8.0 (5.4–9.2)	76.0 (75.5–76.9)	0.92 (0.85–0.97)	51.3 (49.1–53.4)
1.5 ‘extent of peat above flood limit’	1.6 (1.5–1.8)	61.4 (54.2–67.9)	6.2 (6.0–6.5)	5.5–7.1 (4.7–8.2)	76.7 (76.0–77.5)	0.87 (0.80–0.93)	52.8 (51.0–55.0)
2 ‘minimum peat extent’	1.5 (1.3–1.7)	56.1 (49.3–62.1)	6.0 (5.7–6.3)	4.9–6.3 (4.1–7.3)	77.4 (76.5–77.8)	0.82 (0.75–0.88)	54.3 (52.7–56.7)
	Jambi coastal lowland						
0 ‘maximum peat extent’	0.40 (0.34–0.44)	71.8 (61.6–79.3)	6.3 (6.2–6.6)	1.4–1.8 (1.2–2.0)	13.2 (12.8–13.3)	0.19 (0.17–0.20)	48.0 (46.4–50.6)
0.72 ‘most likely peat extent’	0.33 (0.22–0.44)	60.0 (40.5–78.5)	6.2 (6.2–6.5)	1.1–1.4 (0.76–2.0)	12.8 (11.9–13.3)	0.17 (0.13–0.20)	50.9 (46.7–59.3)
1 ‘extent of peat below high tide limit’	0.31 (0.25–0.36)	56.1 (44.5–65.7)	6.1 (6.1–6.2)	1.0–1.3 (0.84–1.6)	12.6 (12.0–13.0)	0.16 (0.14–0.18)	52.5 (49.7–58.1)
1.5 ‘extent of peat above flood limit’	0.26 (0.21–0.32)	47.2 (38.1–57.4)	6.1 (6.1–6.2)	0.88–1.1 (0.72–1.4)	12.1 (11.8–12.6)	0.15 (0.13–0.17)	56.9 (51.9–59.4)
2 ‘minimum peat extent’	0.22 (0.18–0.27)	40.1 (33.1–48.9)	6.2 (6.1–6.2)	0.75–1.0 (0.62–1.2)	11.9 (11.7–12.3)	0.13 (0.11–0.15)	59.4 (55.8–60.1)
	South Sumatra coastal lowland						
0 ‘maximum peat extent’	0.36 (0.32–0.43)	35.5 (31.3–41.9)	6.2 (6.0–6.3)	1.2–1.6 (1.0–1.9)	11.7 (11.5–12.3)	0.10 (0.10–0.10)	28.2 (24.1–31.7)
2.47 ‘most likely peat extent’	0.20 (0.14–0.28)	19.9 (13.8–27.5)	5.3 (4.9–5.7)	0.59–0.75 (0.37–1.1)	10.6 (9.7–11.3)	0.08 (0.07–0.10)	41.6 (34.7–49.6)
1 ‘extent of peat below high tide limit’	0.30 (0.25–0.33)	29.0 (24.7–32.7)	5.8 (5.6–6.1)	0.94–1.2 (0.77–1.4)	11.4 (11.1–11.6)	0.10 (0.09–0.10)	33.5 (30.5–37.2)
1.5 ‘extent of peat above flood limit’	0.26 (0.22–0.30)	25.9 (21.4–29.5)	5.6 (5.5–5.9)	0.81–1.0 (0.65–1.2)	11.2 (10.7–11.4)	0.10 (0.09–0.10)	36.0 (33.1–40.3)
2 ‘minimum peat extent’	0.23 (0.20–0.27)	22.1 (19.2–26.4)	5.5 (5.3–5.7)	0.68–0.87 (0.56–1.1)	10.8 (10.6–11.2)	0.09 (0.08–0.10)	39.6 (35.5–42.3)

Below-ground deep peat carbon stock is calculated based on carbon densities ranging from 0.0545 and 0.0698 g cm^−3^. Deep peat forest cover in 2012 [86] and deep peat area extent relative to [56] are provided as well. Values in brackets provide range taking into account the accuracy of the DTM (RMSE 0.25–0.61 m; [69])

### Deep peat below-ground carbon stock map for the eastern Sumatra provinces

The deep peat maps were used to calculate the below-ground carbon stock in the eastern Sumatra study area. This yielded a minimum and maximum peat volume of 115.9 and 191.1 km^3^, assuming a peat bottom at 2 or 0 m +MSL respectively, and a most likely value of 165.2 km^3^ at a peat bottom of 0.61 m +MSL.

Applying a range in carbon density values of 0.0545 and 0.0698 g cm^−3^ to the deep peat maps, a total peat carbon stock range of 6.3 to 13.3 Pg (applying a peat bottom of 2 or 0 m +MSL, respectively) was determined for the eastern Sumatra lowland area covered by the DTM, with a most likely range of 9.0–11.5 Pg (applying a peat bottom of 0.61 m +MSL). When applying a different most likely peat bottom value for each of the three provinces separately and spatially averaging the result based on deep peat extent in each of the three provinces, the total peat carbon stock ranged from 9.5 to 12.1 Pg, slightly higher than when applying an overall average peat bottom of 0.61 m +MSL. Most of the deep peat carbon stock, i.e. 75.1%, is found in Riau province, with a range of 7.7–9.9 Pg; Jambi and South Sumatra provinces follow at 12.8 (1.1–1.4 Pg C) and 10.6% (0.59–0.75 Pg C), respectively (Table 3).

## Discussion

The most likely deep peat area of 2.6 Mha identified for eastern Sumatra in this study (Table 3) constitutes 39.0% and 17.2% of the total mapped Sumatra and Indonesia peatland area of 6.6 and 14.9 Mha, respectively [56]. If we assume that all remaining peat in Sumatra has a similar thickness distribution to that in the study area, with 60.1% of total peat area (4.3 Mha within the DTM extent of the study area) being deep peat, the total Sumatra deep peat area would be 3.9 Mha and the associated total Sumatra deep peat carbon stock would range between 13.9 and 17.8 Pg. This value is somewhat higher than the high end estimate of 11.9 Pg as reported by [26] using the [56] peat map for both extent and depth (applying the high end of each depth range), for all peat in Sumatra including the shallow peat that we exclude in this study. Whilst we do not have further field data on peat thickness to support an assumption of equal deep peat distribution in other provinces, the distribution of deep peat (> 3 m) in [56] for the Riau, Jambi and South Sumatra provinces is, at 28.7% (1.7 Mha out of a total peat extent of 5.8 Mha), similar to the 26.7% (1.7 Mha out of 6.4 Mha) distribution of deep peat for all provinces in Sumatra combined [56] and as such we consider this a fair assumption. It is furthermore noted that by far the largest extent of deep peat in Sumatra is located in Riau province (93.7% according to [56]), albeit that our area value of 2.1 Mha (Table 3) is some 0.5 Mha higher compared to [56] who mapped 1.6 Mha of deep peat. In addition, while not further mapped in this study, we note that shallow peat also plays a role in carbon storage and release.

Peat that is below the water table, both currently and in the future, will most likely not generate substantial carbon emissions from oxidation and therefore may be excluded from carbon stock calculations used in predicting future emissions [11, 71], although it is noted here that continued DOC loss and shoreline erosion are potential pathways for continued but slower carbon loss from inundated coastal peatlands [87]. In addition, sea water provides abundant sulphate and sulphate reduction that can oxidize organic matter in anaerobic environments [88]. The extent to which this would happen in Indonesian coastal peatlands subject to sea water incursion and inundation would, however, depend on the composition of the organic material as anaerobic bacteria may not be able to decompose the complete suite of organic compounds [88].

Along the East Sumatra coastline, the land surface below 1 m +MSL is currently below the common high tide level, usually covered by permanent swamps and mangroves in their natural state, and most land below 1.5 m +MSL is already inundated much of the time. Peat deposits at these low elevations are, therefore, an unlikely source of future carbon emissions. When accounting for sea level rise, even a low estimate increase of 0.52 m by 2100 [89], will likely mean that only peat at elevations above 1.5 m or 2 m +MSL will contribute substantially to future carbon emissions. The total volume of the deep peat carbon stock above the 1.5 m +MSL reference level ranges between 7.2 and 9.3 Pg (Table 3) and this can be considered the most at risk.

### Past peat loss and the need to monitor peat thickness

The map of thickness of deep peat provided in this study applies to 2017, when most of the airborne LiDAR data used for the underlying land surface elevation model (DTM) were collected [69]. However, continuous peat loss from oxidation resulting in land surface subsidence currently occurs in nearly all peatlands in Sumatra and Kalimantan, by rates varying from just below 2 cm year^−1^ in forested peatland that is a few kilometres away from canals to over 4 cm year^−1^ in actively drained plantations with water levels lowered to an average of around 0.7 m below the peat surface [29, 90]. Fires on peatland are a further source of subsidence by up to 0.3 or 0.5 m for a single event [36, 91–93]. Over periods of 10 to 20 years, cumulative peat loss subsidence will therefore exceed 0.5 m in most remaining peatlands in the region.

**Fig. 10:**
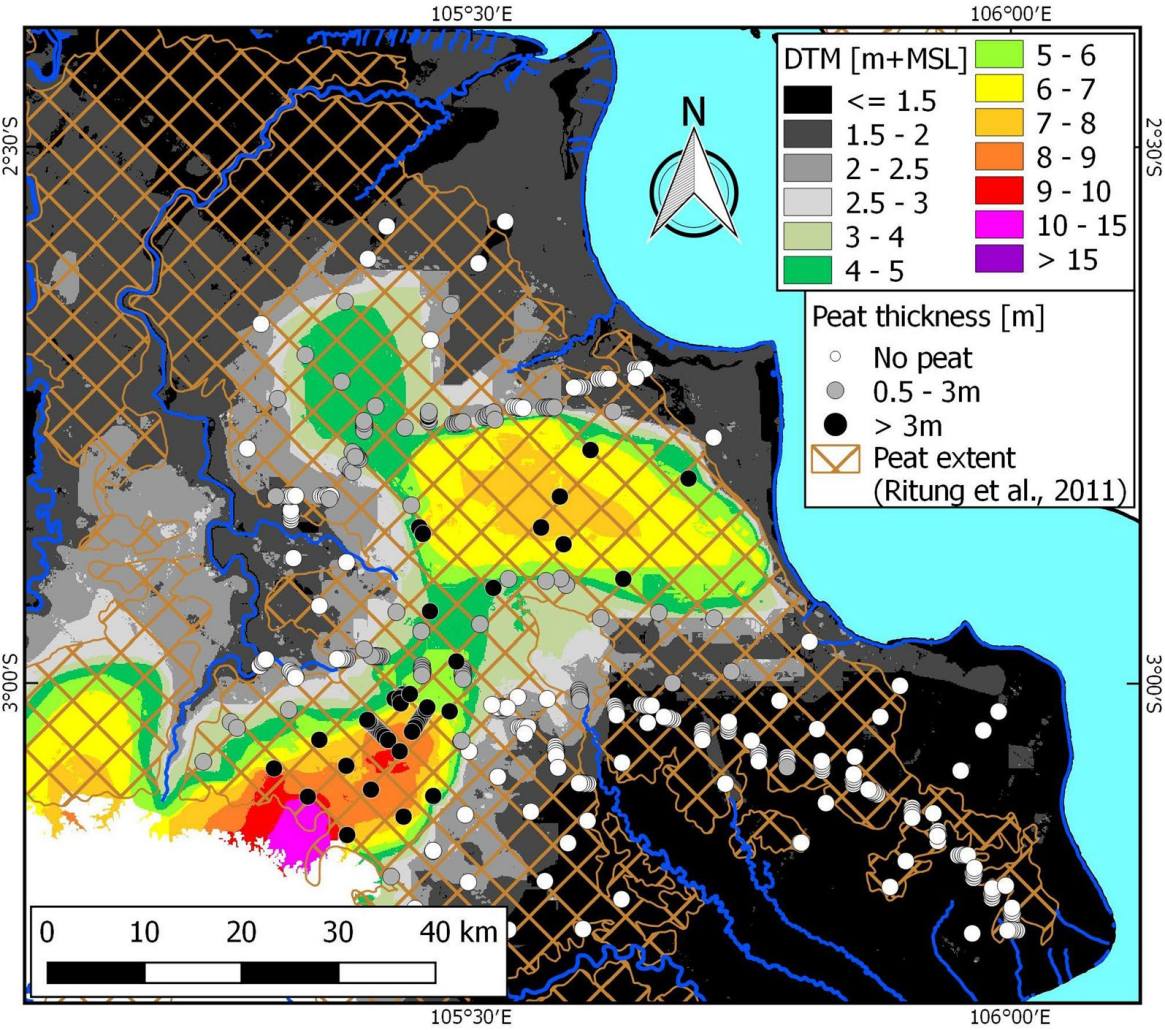
Map of land surface elevation in the Eastern part of South Sumatra, and peat extent [56]. Grey dots indicate locations where peat thickness measured between 2013 and 2016 is less than 3 m and black dots greater than 3 m. White dots are locations without peat

The resulting uncertainty in peat extent and thickness created by ongoing peat loss is well illustrated for the Ogan Komering Ilir landscape in South Sumatra. The forest in this area was completely removed before 2000 [25], mostly in the 1980s [94], and subsequently drained for agriculture and forest plantations. A central peat dome is visible from the 2017 DTM (Fig. 10). However, where [56] mapped most of the area as peat, field surveys over 2013–2016 found only localized pockets of shallow peat in the low lying area outside of one remaining larger dome (Fig. 10). A combination of peat decomposition and repeated fires has resulted in peat loss, such that the land surface in most of the area is now below 2 m +MSL (Fig. 10).

For practical applications peat thickness mapping should, in effect, be seen as peat thickness monitoring. For example, in areas where the peat surface is already low enough to cause flooding problems, regular updates of DTMs and associated peat thickness maps could be used to support flood risk assessment and land use planning decisions. Peat thickness monitoring can also provide information on carbon emissions caused by peat loss [29]. As any peat thickness map can, however, be outdated in a matter of years, the maps should be updated regularly, while accepting that the accuracy of peat thickness maps may never be very high in conditions where peat continues to be rapidly lost. Once an initial peat thickness map has been created from a DTM and sufficient peat thickness measurements have been obtained to estimate the peat bottom level, subsequent revised maps can be generated relatively easily from updates of the DTM.

### Deep peat areas and forest cover in eastern Sumatra

**Fig. 11:**
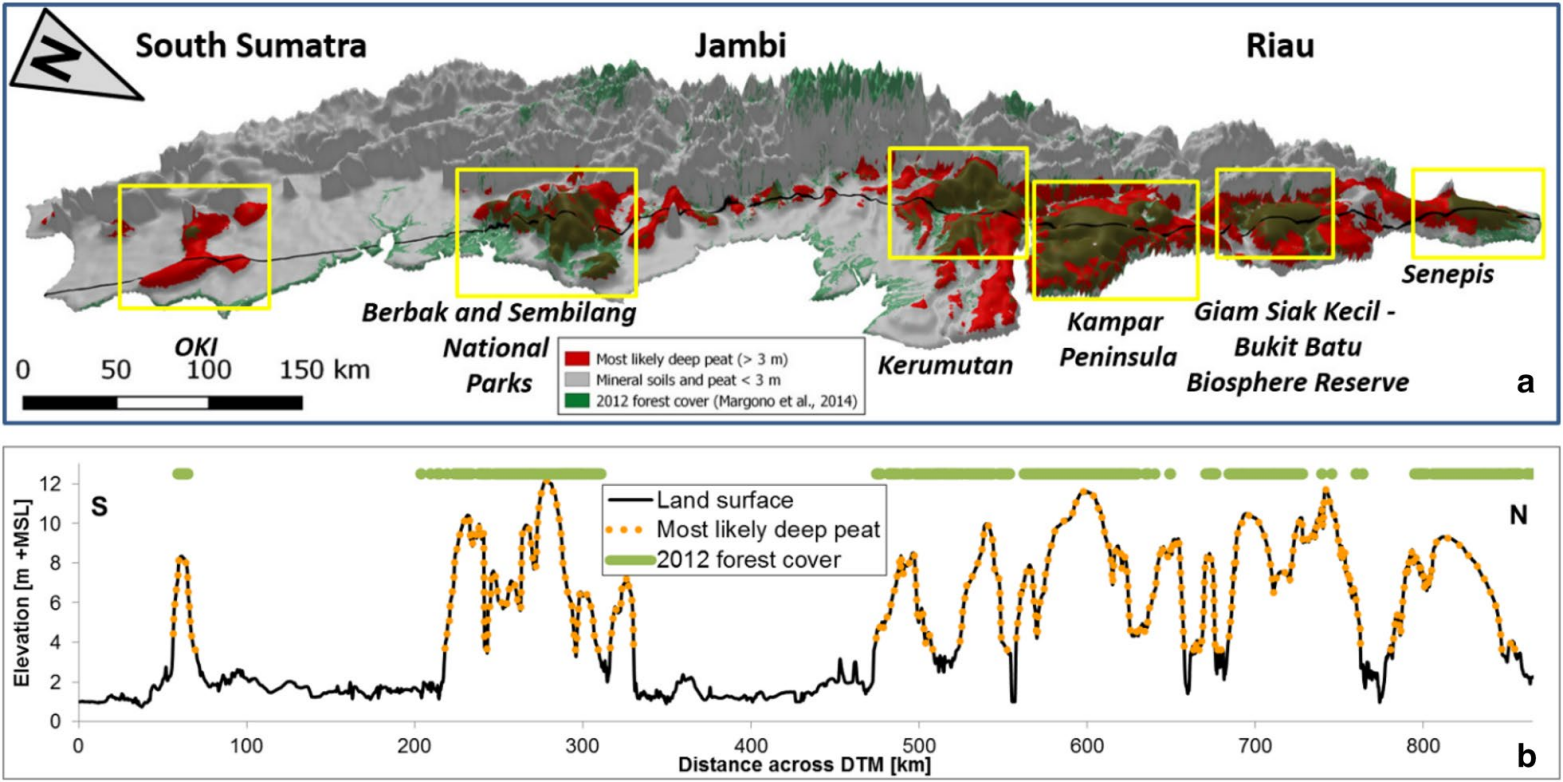
**a** 3D model of eastern Sumatra DTM (Fig. 1) superimposed with the modelled deep peat areas and 2012 forest cover [86]. The airborne LiDAR based lowland DTM was merged with a SRTM based DTM for upland areas, to show the full landscape; elevations above 10 m +MSL have sharply reduced vertical scale, by a factor 5. Shown as well the location of surface elevation profile (black line) shown in (**b**) Surface elevation cross section along entire eastern Sumatra DTM, at approximately 20–70 km from the coastline. Most likely deep peat surface (> 3 m) is shown assuming a peat bottom at 0.61 m +MSL. Indicated are the six major peat domes along the East Sumatra coast, from North to South: Senepis, Giam Siak Kecil—Bukit Batu Biosphere Reserve, Kampar Peninsula, Kerumutan in Riau, Berbak and Sembilang National Parks in Jambi and South Sumatra and OKI in South Sumatra Province. Note that the peat swamp forest on Kampar Peninsula and Senepis are not formally protected

Apart from a large below-ground carbon stock, in 2012 the deep peat areas also hosted nearly all (1.3 Mha or 93.6%) of the remaining non mangrove forest in the DTM study area [86], as is illustrated in Fig. 11. In fact, large areas of intact forest remain on a few major peat domes (Fig. 11). Parts of these forested peat domes are formally protected and designated National Parks or Wildlife Reserves [59], but none are fully protected and all are under threat from logging and fires along their boundaries [58, 95]. Protecting deep peat areas therefore currently still provides an opportunity to not only conserve large amounts of below-ground carbon stock, but also the last Sumatra lowland forest and associated high above-ground carbon stocks and unique biodiversity [96].

### Cost effective peat mapping using elevation models

**Fig. 12:**
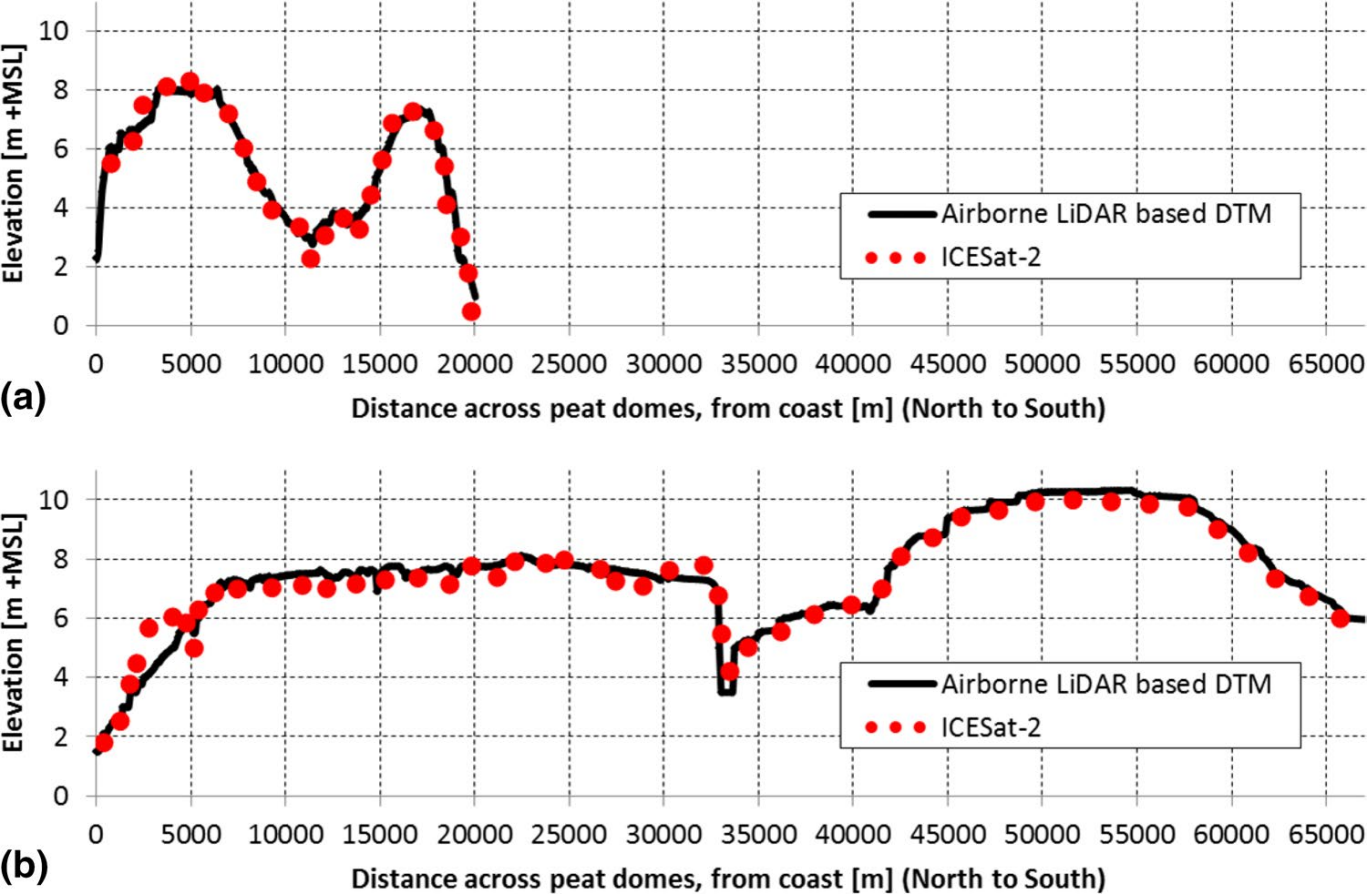
Cross section over **a** Bengkalis Island and **b** the Giam Siak Kecil—Bukit Batu Biosphere Reserve covering peat domes along ICESat-2 flight lines, showing airborne LiDAR derived surface elevation (DTM; [69] and ICESat-2 terrain height. As current raw ICESat-2 data is referenced to the ellipsoid and requires vertical correction, it was provisionally referenced to mean sea level (MSL) by matching it to the DTM. Location of the cross sections is shown for **a** in Fig. 4 and **b** in Fig. 1

The deep peat map presented in this paper was generated using an elevation model derived from interpolated partial coverage airborne LiDAR data [69]. The associated cost of the airborne LiDAR data acquisition and processing was 1.5 USD per ha of actual LiDAR coverage [69], although substantially less for DTM coverage based on partial LiDAR coverage. For application at the very large scale of countries and regions, requiring coverage of tens of millions of hectares, this high cost is limiting progress in peat mapping. However high resolution elevation mapping is expected to rapidly become very cost effective with satellite LiDAR data from the Advanced Topographic Laser Altimeter System (ATLAS) on board the Ice, Cloud and land Elevation Satellite (ICESat)-2 [97] and the Global Ecosystem Dynamics Investigation (GEDI) LiDAR attached to the International Space Station (ISS) [98, 99] being collected since late 2018 with the first data of the former available to the public by mid-2019, without cost [97]. The potential of ICESat-1 data for measuring peat topography was earlier demonstrated by [100]. Our analysis of first ICESat-2 data reveals peat dome shapes that are identical to the existing eastern Sumatra DTM used in this study as shown in Fig. 12. By late 2021, ICESat-2 data alone is targeted to provide near-global coverage (between 88° north and south latitudes; [97]) of N–S oriented flight lines at intervals of less than 2 km at the equator [97, 101], with GEDI expected to provide additional coverage with a spacing between tracks of about ~ 600 m [99] between 51.6° north and south latitudes [102]. This will allow creation of landscape DTMs as demonstrated by [69], that are suitable for rapid global mapping of peat domes applying the method presented in this paper, and thereby improving estimates of regional and global peat carbon stocks and their vulnerability.

## Conclusions and recommendations

This paper demonstrates that it is possible to create a peat thickness map that is suitable for the purposes of land use zoning and carbon stock calculations from a LiDAR-derived DTM and with limited peat thickness field measurements, in raised bog type peatland with relatively uniform peat bottom elevation. In areas where the peat bottom is more variable, or in conditions where accuracy requirements are very high, more peat thickness measurements can be added stepwise until certain accuracy criteria are met. However, where the peat bottom is around or below 1.5 m +MSL in SE Asian lowlands, it may be considered whether high accuracy in peat thickness mapping is required as this peat is expected to be permanently below the water table due to sea level rise. These low-lying peatlands may not, therefore, generate substantial carbon emissions in the future, albeit we note the potential for a positive carbon feedback to the atmosphere from inundated coastal peatlands [87].

We believe that the map of deep peat in eastern Sumatra presented in this study can make a contribution to broader approaches to peat mapping in Indonesia. Where higher accuracy is required, the map may be refined by collecting additional data. Mapping shallow peat areas will require more ground data or possibly geophysical methods, such as ground-penetrating radar (GPR) and electrical resistivity imaging [103], although it is noted that GPR also has its limitations when saline groundwater is present in shallow aquifers of coastal peatlands, because saline water attenuates GPR signals [104]. Both the DTM [69] and the deep peat map used in this analysis are available in the public domain from (10.17632/c83z4df8ky.1) to be used in application and further analysis.

By focusing on remaining deep peat in SE Asia, much of which is still forested, it will be easier to define and implement strategies to protect the remaining peatland. The deep peat carbon stock range of 9.0–11.5 Pg above the 0.61 m +MSL average peat bottom reference level in the eastern Sumatra study area alone, represents 8.6–11.0% of the 104.7 Pg global tropical peat carbon stock [2, 21], in a relatively small land area of 2.6 Mha that is the equivalent of Belgium or Wales. We propose that in the SE Asian context, prioritizing the protection of deep peat areas and associated forest could result in the best conservation outcomes both in terms of reduced carbon emissions and the safeguarding of forest and biodiversity. Nevertheless, ongoing peat loss resulting from the rapid pace of land use change (scale and intensity) and the regular occurrence of fires across the whole of the SE Asian region will necessitate regular updating of peat maps to ensure that they remain relevant.

The peat mapping method described here is applicable to any peatland that has a somewhat flat peat bottom. This includes some ‘raised bogs’ located in shallow basins and found in coastal lowlands and alluvial plains throughout continental Eurasia and North America, but could also extend to peatlands in the Congo and Amazon river basins as well, although knowledge of the morphology of these peatlands is still under investigation [105]. The method would exclude peatlands formed in deep basins (e.g. in kettle holes) as well as most upland northern peat deposits that tend to be ‘blanket bogs’ that follow a pre-existing landscape. We therefore propose rapid application of this method using satellite LiDAR data, complemented with airborne LiDAR where available, to other ‘raised bog’ peatlands globally that require improved peatland mapping.

## Data Availability

The DTM (10.17632/72r3bvdd3r.1) and the deep peat map (10.17632/c83z4df8ky.1) used in this analysis are available in Mendeley.

## Abbreviations

ATLAS: Advanced Topographic Laser Altimeter System
BD: Bulk density
CC: Carbon concentration
DTM: Digital Terrain Model
GEDI: Global Ecosystem Dynamics Investigation
ICESat: Ice, Cloud and land Elevation Satellite
IPP: Indonesian Peat Prize
LiDAR: Light Detection and Ranging
MSL: Mean sea level
PB: Peat bottom
PT: Peat thickness
